# Case report: Tolosa-Hunt syndrome—expanding the neuromyelitis optica spectrum disorder phenotype?

**DOI:** 10.3389/fneur.2024.1326867

**Published:** 2024-02-14

**Authors:** Soo-Hyun Park, Soo-Im Jang, Eun-Ja Lee, Nam-Hee Kim

**Affiliations:** ^1^Department of Neurology, Soon Chunhyang University Hospital, Seoul, Republic of Korea; ^2^Department of Neurology, Dongguk University Ilsan Hospital, Goyang, Republic of Korea; ^3^Department of Radiology, Dongguk University Ilsan Hospital, Goyang, Republic of Korea

**Keywords:** optic neuritis, neuromyelitis optica spectrum disorder, Tolosa-Hunt syndrome, abducens palsy, aquaporin-4 antibody, AQP4-immunoglobulin G

## Abstract

Neuromyelitis optica spectrum disorder (NMOSD) is an autoimmune astrocytopathy caused by the autoantibody of aquaporin-4 (AQP4). Herein, we report a case of Tolosa-Hunt syndrome presenting with abducens palsy and AQP4 antibodies. This was a rare case of AQP4-immunoglobulin G seropositivity in a patient with Tolosa-Hunt syndrome. Our findings may expand the clinical phenotype of NMOSD and indicate that clinicians should consider testing for AQP4 antibodies in patients with Tolosa-Hunt syndrome.

## 1 Introduction

Neuromyelitis optic spectrum disorder (NMOSD) is an autoimmune, demyelinating disease of the central nervous system (1). The autoantibody of aquaporin-4 (AQP4), a diagnostic biomarker, is detected in up to 80% of patients with NMOSD who meet the clinical and radiologic criteria (2, 3). NMOSD presents with syndromes or lesions of the optic nerve, spinal cord, brainstem, thalamus/hypothalamus, area postrema, and cerebrum, which are defined as the core clinical characteristics (1). Tolosa-Hunt syndrome is characterized by steroid-responsive painful ophthalmoplegia from idiopathic granulomatous inflammation of the cavernous sinus, superior orbital fissure, or orbit (4). To our knowledge, Tolosa-Hunt syndrome has rarely been reported in patients with NMOSD (5). This report describes a case of Tolosa-Hunt syndrome with AQP4 antibody seropositivity.

## 2 Case report

**Figure 1:**
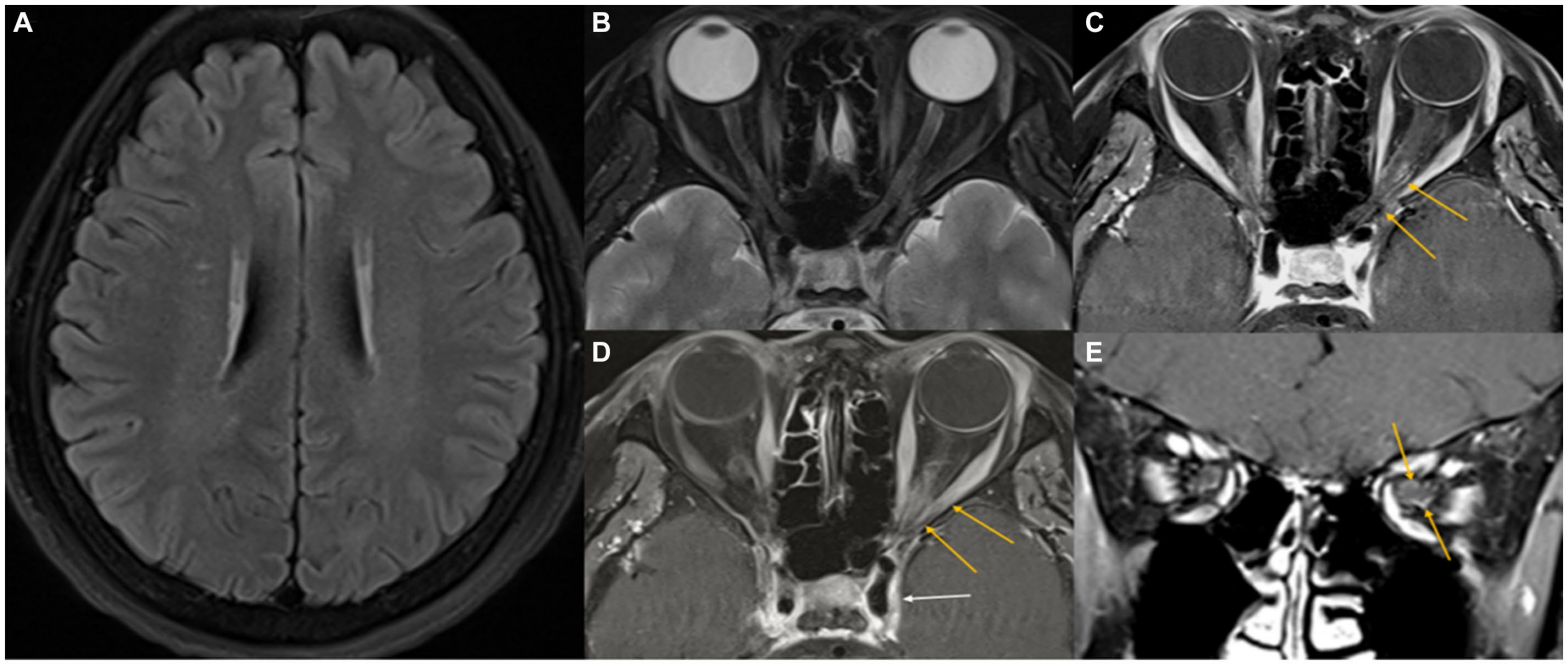
Initial MR images. Axial T2-weighted FLAIR image showing several small high-signal foci in the cerebral white matter **(A)**. A fat-suppressed axial T2-weighted image shows a slight infiltrative change in the left orbital apex with intermediate signal intensity **(B)**. Fat-suppressed contrast-enhanced axial and coronal T1-weighted images reveal mild perineural enhancement in the left orbital apex (yellow arrows), with no enhancement of the optic nerve **(C–E)**. A suspicious extension into the left cavernous sinus through the superior orbital fissure (white arrow) is noted.

**Figure 2:**
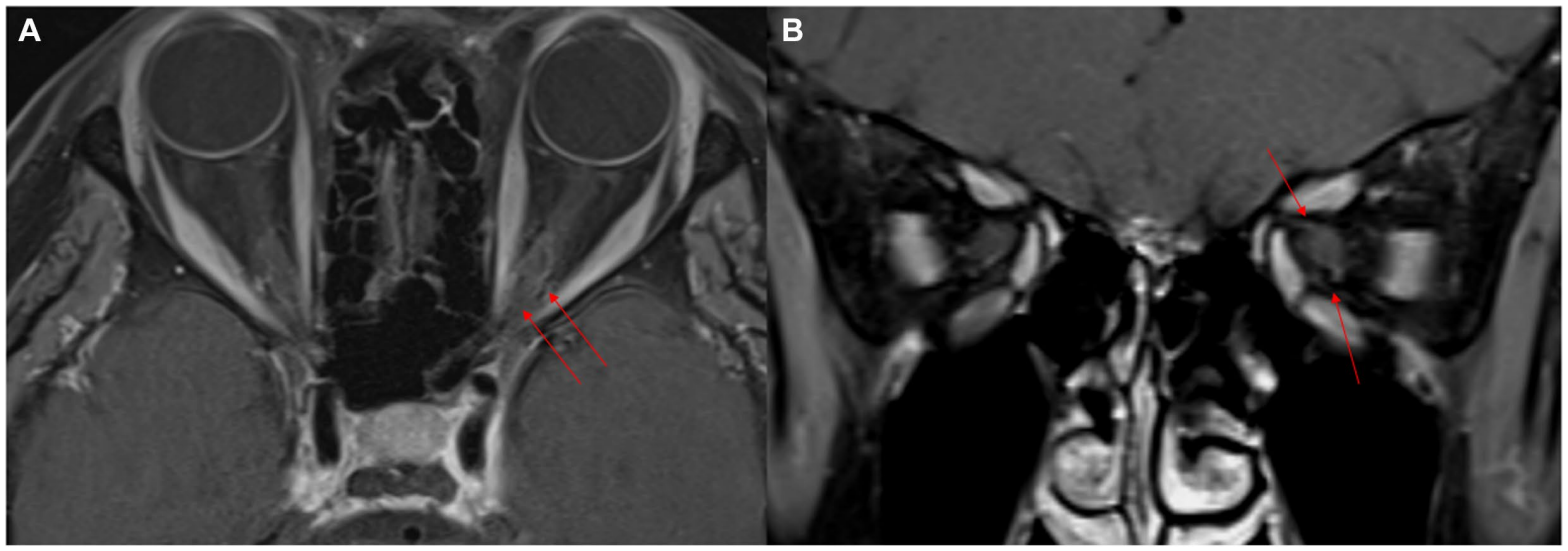
Two-month follow-up MR images after steroid pulse therapy. Fat-suppressed contrast-enhanced axial and coronal T1-weighted images show improved perineural enhancement in the left orbital apex (red arrows). The size of the left cavernous sinus appears to have reduced (red arrows) **(A,B)**.

A 53-year-old previously healthy woman presented with acute-onset binocular horizontal diplopia that was aggravated when looking to the left, with dull periorbital pain and subtle visual blurring in the left eye. She had no history of fever, nausea, nasal congestion, rhinorrhea, or conjunctival injection, and no significant medical history. Neurological and ophthalmological examination revealed a left abducens nerve palsy. The visual acuity was 1.0 and 0.8 decimal in the right and left eye, respectively, with normal findings on fundoscopy and optical coherence tomography, and visual evoked potential study. Brain and orbital magnetic resonance imaging (MRI) demonstrated gadolinium enhancement of the left cavernous sinus, perineural enhancement of the optic nerve at the left orbital apex, and a few nonspecific high signal intensities in the periventricular white matter (Figure 1). Whole spine MRI presented no abnormalities in the spinal cord. Cerebrospinal fluid analysis revealed no pleocytosis (1 white blood cell/μL), normal protein levels (32.8 mg/dL), and the absence of oligoclonal bands. Laboratory tests revealed no abnormalities, including erythrocyte sedimentation rate, C-reactive protein, serum protein electrophoresis, blood cell count, thyroid function, angiotensin-converting enzyme, antinuclear antibodies, antineutrophil cytoplasmic antibody, rheumatoid factor, Sjogren-related antibodies, antibodies against acetylcholine receptor antibody (binding), GM1, GQ1b, IgG4, and myelin oligodendrocyte glycoprotein (MOG)-IgG. However, elevated anti-aquaporin 4 IgG levels in the serum (titer 1:10) were detected using an indirect immunofluorescence assay. Although the patient presented with subtle visual impairment, she was diagnosed with NMOSD, considering mild optic neuritis with AQP4 antibody positivity. She was treated with IV steroid pulse therapy (methylprednisolone, 1,000 mg/day for 5 days), followed by 60 mg of oral prednisone daily. Her pain resolved and gradually improved both clinically and radiologically (Figure 2). At 1 month, the horizontal diplopia had completely improved, and the visual acuity in the left eye had changed from 0.8 to 1.0 decimal. A repeat AQP4-IgG test result was negative, together with improvement in the MRI abnormality at 2 months. The patient had no recurrence in the subsequent 2 years on azathioprine (100 mg daily).

## 3 Discussion

To our knowledge, this is the first report of Tolosa-Hunt syndrome with AQP4-immunoglobulin G seropositivity. Our patient, who had mild optic neuritis and was seropositive for AQP4-IgG, presented with symptoms of horizontal diplopia and pain as well as inflammation of the cavernous sinus and orbital apex, leading to a clinical diagnosis of Tolosa-Hunt syndrome. These features have not previously been recognized in AQP4-IgG-related disorders. Therefore, our findings may expand the clinical spectrum of NMOSD phenotypes.

Recent NMOSD diagnostic criteria include the following: (i) at least one core clinical characteristic, (ii) positive test for AQP4-IgG, and (iii) exclusion of alternative diagnoses (3, 6). Although the optic nerve did not present the long segment involvement typical of NMOSD, the diagnosis was considered as probable NMOSD because our patient met the criteria for optic neuritis and was seropositive for AQP4 antibody. In our case, abduction palsy with cavernous and orbital apex inflammation is an uncommon symptom in NMOSD.

Tolosa-Hunt syndrome is characterized by idiopathic orbital inflammatory diseases, including orbital pseudotumors (7), and is associated with cranial nerve palsy (4, 8). The ocular motor cranial nerves (III, IV, and VI), optic nerve (II), and infrequently other cranial nerves (V and VII) or sympathetic innervation of the pupil may be involved (4, 9). The current patient exhibited involvement of the optic (II) and abducens (VI) nerves and periorbital pain, which was consistent with the typical clinical features of Tolosa-Hunt syndrome. MRI also revealed inflammation in the cavernous sinus and orbital apex, supporting the diagnosis of Tolosa-Hunt syndrome.

Tolosa-Hunt syndrome has diagnostic criteria but is a diagnosis of exclusion. An extensive workup is required for the wide differential diagnoses of pai0nful ophthalmoplegia (4, 10). Systemic diseases, such as IgG4-related disease, granulomatosis with polyangiitis, polyarteritis nodosa, and sarcoidosis, are known to present a clinical picture similar to that of noninfectious orbital inflammation (7). To date, AQP4 antibodies have not been detected in Tolosa-Hunt syndrome, whereas MOG antibodies have recently been detected in orbital pseudotumors with ON (11, 12).

Nonspecific granulomatous inflammation of the cavernous sinus wall is a pathological finding in Tolosa-Hunt syndrome. It extends through the superior orbital fissure, the optic canal, or the inferior orbital fissure (6). Since the cavernous sinus is surrounded by the dura and idiopathic pachymeningitis may be a manifestation of peridural inflammation, Tolosa-Hunt syndrome may be a clinical presentation of idiopathic pachymeningitis affecting the cavernous sinus (13). Tolosa-Hunt syndrome and pachymeningitis, at least in part, have a common cause, and they may comprise a disorder caused by “idiopathic inflammation of the dura mater” (13–15). Cranial nerve involvement has been observed in several cases of hypertrophic pachymeningitis have been recognized with cranial nerve involvement (4). Although no focal cavernous sinus lesions have been reported in NMOSD to date, a cavernous sinus lesion due to focal pachymeningitis may reasonably be assumed to result from NMOSD (5).

In particular, abduction palsy with cavernous and orbital apex inflammation is not easy to explain by NMOSD. However, hypertrophic pachymeningitis has also occurred in NMOSD without and with peripheral cranial nerve involvement (5, 14–17). In previous reports, some painful idiopathic pachymeningitis with multiple cranial neuropathy demonstrated asymmetric cavernous sinus enhancement on brain MRI, similar to Tolosa–Hunt syndrome. Therefore, it seems reasonable to assume that a cavernous sinus lesion by focal pachymeningitis could occur because of NMOSD. This case provides physicians with a broader perspective on the possibility of NMOSD in patients with Tolosa-Hunt syndrome or pachymeningitis.

This case provides physicians with a broader perspective on the possibility of NMOSD in patients with Tolosa-Hunt syndrome or pachymeningitis. This case will help expand our knowledge of the clinical phenotypes of NMOSD. If similar cases are found in the future, it may be necessary to consider adding an AQP4 antibody test, which is not currently performed in patients with Tolosa-Hunt syndrome or pachymeningitis.

## Data availability statement

The raw data supporting the conclusions of this article will be made available by the authors, without undue reservation.

## Ethics statement

The studies involving human participants were reviewed and approved by the Ethics Committee of Dongguk University Ilsan Hospital. The patients/participants provided their written informed consent to participate in this study. The studies were conducted in accordance with the local legislation and institutional requirements. Written informed consent was obtained from the individual(s) for the publication of any potentially identifiable images or data included in this article.

## Author contributions

S-HP: Conceptualization, Formal analysis, Writing – original draft, Writing – review & editing. S-IJ: Conceptualization, Formal analysis, Investigation, Writing – original draft. E-JL: Conceptualization, Data curation, Formal analysis, Writing – original draft. N-HK: Conceptualization, Formal analysis, Investigation, Supervision, Writing – original draft, Writing – review & editing.
